# Five-Year Data on Pollen Monitoring, Distribution and Health Impact of Allergenic Plants in Bucharest and the Southeastern Region of Romania

**DOI:** 10.3390/medicina55050140

**Published:** 2019-05-15

**Authors:** Polliana Mihaela Leru, Ana-Maria Eftimie, Vlad Florin Anton, Michel Thibaudon

**Affiliations:** 1 Carol Davila University of Medicine and Pharmacy, 050474 Bucharest, Romania; 2 Colentina Clinical Hospital, 020125 Bucharest, Romania; anamariaeftimie@yahoo.com (A.-M.E.); antonvladflorin@gmail.com (V.F.A.); 3 Réseau National de Surveillance Aérobiologique, 69690 Brussieu, France; michel.thibaudon@wanadoo.fr

**Keywords:** aerobiology, *Ambrosia*, biologic pollution, pollen monitoring, respiratory allergies

## Abstract

*Background and objectives:* Respiratory allergies induced by allergenic pollen represent an important public health problem with increasing prevalence and severity in Europe. Romania has no aerobiology network and pollen measurements have been done for about ten years in the west region only. *Materials and Methods:* We established the first pollen monitoring center in the capital of Bucharest in 2013, based on collaboration with the Réseau National de Surveillance Aérobiologique (RNSA) from France. The aim of our paper is to present results from five years of pollen monitoring in the city center of Bucharest and preliminary data on distribution and health impact of some allergenic plants, mainly *Ambrosia artemisiifolia*, which is considered a real danger for the public health. *Results:* Our data show a significant atmospheric amount and a longer season than previously considered of grass (*Gramineae*) pollen and short period with a high level of *Ambrosia* pollen, while tree pollen looks less important in this area. The plant distribution data provided by specialists and information from affected persons showed the wide and increasing spread of *Ambrosia* in Bucharest and other cities from the south region. Preliminary health data from allergists confirmed that the number of patients with allergies to *Ambrosia* pollen is increasing from one year to another and almost all patients describe a high urban exposure from their living or working place. *Conclusions:* We consider that the recently implemented Law 62/2018 against *Ambrosia* may help reduce weed distribution and the atmospheric pollen load, but a more complex and coordinated strategy for controlling urban vegetation and reducing biologic pollution is needed.

## 1. Introduction

Allergic diseases, including asthma, are considered a prominent cause of disability and mortality in the world, with major social and economic consequences [1]. A recently published 25-year population study demonstrated a continuous increase of respiratory symptoms and diseases, including asthma and allergic rhinitis, in European countries [2]. Respiratory allergies induced by allergenic pollen represent an important public health problem, with increasing prevalence and severity in Europe over the last few decades [3]. Pollen grains from anemophilous plants and molds are important bioparticles of the atmosphere. Recently published data revealed regional differences between countries and some changes in the pollen map of Europe, pointing out the need for global evaluation of the problem [4]. The first pollen monitoring with medical purposes in the world was performed by Blackley in 1870 [5] and the oldest continuous pollen record dates from 1943 in England, where a Hirst pollen trap was used since 1954 [6]. The aerobiological study of allergenic pollens is performed on a regular basis in most European countries and correlated with health data from allergists in the frame of national aerobiology networks. Romania has no national aerobiology network and constant pollen measurements have been done between 1999–2010 in the west region only, in Timișoara city [7]. Despite a significant amount of research and scientific literature published by many specialists in the field of allergenic plants, no pollen measurement data are available for other regions of the country, including the capital of Bucharest. The interest to develop the first aerobiology laboratory in Bucharest came from local allergists in 2009, due to increasing number of patients with respiratory allergies and the need to extend investigations of allergenic pollens from the urban environment. In 2012 Romania was invited for the first time to take part in a COST (European Cooperation in Science & Technology) project dedicated to ragweed, entitled Sustainable Management of Ambrosia artemisiifolia in Europe (SMARTER) FA1203, due to the clear extension of this invasive weed to Eastern European countries and the need for global evaluation of its danger [8]. Based on collaboration with European specialists, we have inaugurated the first aerobiology laboratory in one of the oldest and largest multidisciplinary university hospitals in the capital. The aim of our paper is to present the results of the five years of pollen monitoring in Bucharest between 2014–2018 and preliminary data on the distribution and health impact of some allergenic plants in the capital and Southeastern region of Romania. We paid special attention to *Ambrosia artemisiifolia,* which is considered a real and increasing danger in this area.

## 2. Materials and Methods

### 2.1. Pollen Monitoring in the City of Bucharest

In 2013, in the frame of the COST project SMARTER, we established a long-term collaboration with the Réseau National de Surveillance Aérobiologique (RNSA) from France and in 2014 we started to perform pollen monitoring on a regular basis in Bucharest. We used a Hirst-type volumetric pollen trap placed at a height of 19 m h, on the roof of the four-level building of the Research Development Pavilion at Colentina Clinical Hospital, situated close to the city center. We applied the standardized pollen monitoring method according to the European Aerobiology Society (EAS) recommendations [9]. Monthly results during five consecutive pollen seasons 2014–2018 have been initially sent for validation to the RNSA laboratory and then to European Aeroallergen Network (EAN). Our monthly pollen data have been posted on www.polleninfo.org, giving information about pollens from Bucharest for the first time and the pollen monitoring results of the first three years were published in 2017 [10]. Our attention was focused on grass and ragweed pollen, which are known to be some of the most prevalent allergenic pollen species in this area.

### 2.2. Distribution of Allergenic Plants in Bucharest

We collected data about urban vegetation from specialists and also information and pictures with *Ambrosia* in the proximity of living and/or working places from patients diagnosed with allergies induced by this pollen.

### 2.3. Evaluation of Ambrosia Pollen Induced Allergies

In order to assess the number of patients with allergies induced by *Ambrosia* pollen in Bucharest and other cities from the Southeast region of Romania, we initiated a study based on a questionnaire addressed to allergists, during a period of six months, between November and April 2017. We addressed the questionnaire either directly or via the internet, targeting the allergist members of the Romanian Society of Allergology and Clinical Immunology (RSACI). The RSACI has a total number of 180 members, 165 being allergists. There are 50 allergists registered in Bucharest and another 23 allergists working in six big cities from the region, situated at an average distance of about 100 km from the capital. The main issues were total number of patients with confirmed sensitization to ragweed, new annual cases, possible occupational exposure, place of exposure to Ambrosia, duration and clinical type of allergy, disease severity, mono- or polysensitization, history of atopy or other concomitant allergies, treatment and disease outcome.

## 3. Results

### 3.1. Pollen Monitoring Data in Bucharest between 2014 and 2018

**Figure 1:**
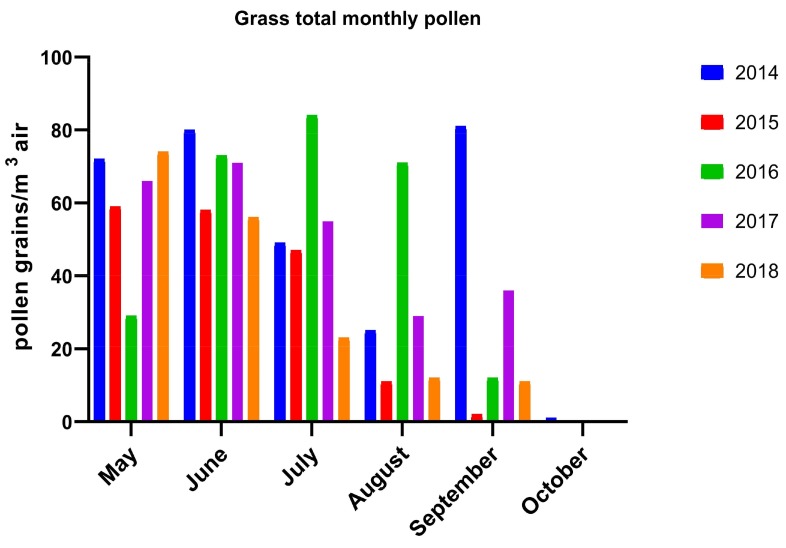
Grass total monthly pollen grains/m^3^ air during the past five years (2014–2018). (Observation: data corresponding to August and September 2015 were distorted by some technical problems of the pollen trap).

**Figure 2:**
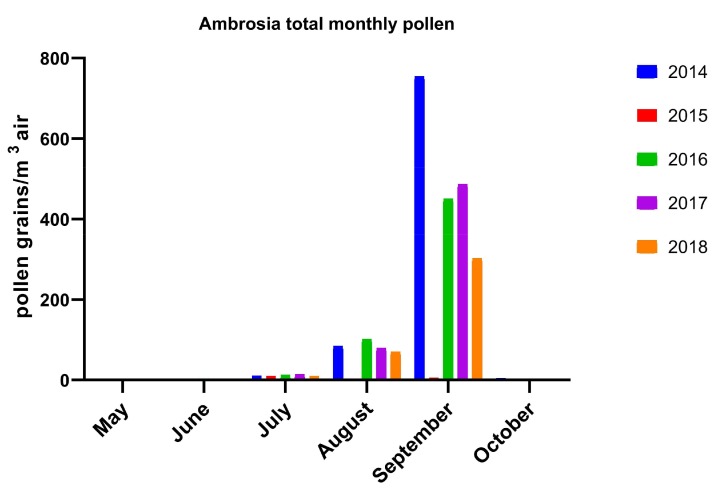
*Ambrosia* total monthly pollen grains/m^3^ air during the past five years (2014–2018). (Observation: data corresponding to August and September 2015 were distorted by some technical problems of the pollen trap).

The pollen monitoring season started in March, except the year 2018, when it started later, due to a longer winter in Romania and consistent amount of snow until end of March in Bucharest, leading to a delayed flowering period. The first recorded pollen species in early spring belong to trees, with relatively abundant silver poplar (*Salicaceae* family), cypress (*Cupressaceae*) and elm (*Ulmaceae*) in March, and mulberry (*Moraceae*) in April, with some of them being not highly allergenic trees (Table 1). In 2016, we recorded very low pollen counts for mulberry and cypress and delayed records for alder, poplar and elm, possibly due to sudden temperature variation, rapid seasonal changing and the shorter duration of spring. The same explanation may apply for missing *Platanus* pollen during 2016 and 2018. Referring to the allergenic trees birch and alder (*Betulaceae* family), we found slightly increased values in March, confirming that they are not relevant in this area, representing more important allergenic pollen species in Central and Northern Europe. During the five years of pollen monitoring in Bucharest, we recorded a high amount of some allergenic species, mainly grass and weeds, the last being represented by *Ambrosia artemisiifolia* (Amb) and *Artemisia vulgaris* (Art). We found a relatively high amount of grass (*Gramineae* family) pollen during May–June and surprisingly in September also, with total monthly pollen values ranging from 25 to 89 grains/m^3^ air (Figure 1). Regarding weed pollen, *Ambrosia* was very abundant during the 2014 season, starting in August and increased significantly during September 2014, with a daily peak value of 231 pollen particles/m^3^ air in early September 2014, to a total monthly amount of 754 grains/m^3^ air and a slightly lower levels during the next years (Figure 2).The pollen count of *Ambrosia* during August and September 2015 was compromised due to technical problems of the trap, therefore we have no reliable data from the summer of 2015. 

### 3.2. Preliminary Data on Distribution of Allergenic Plants in Bucharest

Romania has a temperate-continental climate, with four seasons and an approximate eight months vegetation period. Bucharest is situated at 44° latitude and 26° longitude, in the Romanian Plain, at a maximum altitude of 90 m above sea level and 70 km North from the Danube river. It has an area of 228 km^2^, with 70% building area and a population of almost 1,900,000 inhabitants. The main allergenic plants reported in this area are grass species, which pollinate during spring-summer, and weeds which pollinate in late summer-autumn (Table 2).

Information and pictures collected from patients diagnosed with allergies induced by *Ambrosia* pollen have showed many weed infested areas in all city districts, from the center to periphery, very close to high traffic and crowded centers. The main *Ambrosia* infested places are abandoned construction sites and new residential districts with deficient infrastructure, attributable to the rapid city development and economic crisis of the last ten years.

### 3.3. Preliminary Data on Health Impact of Ambrosia in Bucharest

A total of 16 out of 50 allergists from Bucharest responded to our questionnaire, meaning a response rate of 32%, and 7 allergists out of 23 from six other cities of the southeast region, meaning a response rate of 30.43%. About 2500 patients with a confirmed allergy to *Ambrosia* pollen were recorded in Bucharest during the three years from 2014 to 2016 by the allergists who answered and about 1000 patients in other cities. If we consider the response rate, we may assume that the number of recorded patients is about 8000 in Bucharest and more than 3000 in the other six cities from the southern region. Some relevant aspects noticed were: the increased rate of seasonal new cases during the last years was 2.5–3 times, with significant increases during the last year. Almost all allergists reported a number of 2–5 new confirmed cases daily. The most frequent clinical type of allergy was seasonal allergic rhinitis or rhino-conjunctivitis and about 20% of patients were associated with recent-onset asthma. The general profile of sensitized patients was young and active people, living or working in new districts around Bucharest for the past 2–3 years, with a relatively high education and information level. No clear data about mono- or polysensitization and duration of disease could be obtained, but less than 20% of patients received specific immunotherapy, sublingual in most of the cases.

## 4. Discussion

The results of the last five years of pollen monitoring in Bucharest showed a significant amount of allergenic pollens, mainly grass and *Ambrosia artemisiifolia* pollen. The last was previously considered to be more prevalent in the west regions and rural areas. We found that grass pollen in the atmosphere of Bucharest was increased from May to September, with peak values in June, July and September also, meaning a longer period than considered before. However, our results showed a quite low level of grass pollen compared with data from other countries as described in the literature [11]. No grass pollen data for comparison with other regions of Romania and other years could be found. We also found high levels of Ambrosia pollen from August to September, with a daily peak value of 231 pollen particles per m^3^ air in early September 2014. This value is comparable with 292 particles per m^3^ air mentioned in the literature for the Western city of Timișoara in 2009 [12]. These data confirm that *Ambrosia* is actually a relevant allergen for the urban environment and for the Southeast region of Romania also, but a longer monitoring period and correlation with health data from allergists and other components of air pollution are needed.

Regarding the preliminary results of the study evaluating the health impact of *Ambrosia* pollen, our preliminary estimation based on allergists’ recordings showed at least 8000 sensitized patients in Bucharest and more than 3000 in six other cities from the region. We consider that the real number of sensitized patients is much higher, and it has to be evaluated using other recordings, since many patients do not ask specialist advice and may be seen by family doctors or even by pharmacists during the period of symptoms. Our previous paper evaluating allergists’ opinions about the health impact of *Ambrosia* pollen, published in 2015, demonstrated a high interest and concern on this topic, but also some gaps in terms of prevention and public management of the problem [13]. We intend to continue our study in order to have a higher response rate and obtain more precise data from the allergists and also involve general practitioners.

It was proven that urban vegetation can be dangerous and allergenic species need careful evaluation by specialists, since pollen and fungal spores, as principal components of biologic pollution, have detrimental effects on human health [14]. The two components of air pollution—biological and non-biological—have to be equally considered, due to their added and interrelated detrimental effects. Data from the literature have proven that pollen grains have complex effects on the human immune system, influenced by many factors, such as their association with exogeneous particulate material, including diesel exhaust particles [15]. 

The country-wide and rapid spread of *Ambrosia* in big cities confirms the clear need for better control of urban vegetation. *Ambrosia artemisiifolia* is a species of particular concern in Europe, and Romania is included as the most infected country, but control measures are labor intensive and expensive. Local decision-makers, landscapers and architects have to be informed and should consider specialist recommendations regarding allergenic plant species to be avoided and introduce landscape diversity, in order to reduce atmospheric pollen concentration of some species [16]. There are clear benefits of anticipating the potential future distribution and impact under climate change, increasing awareness of the health impact of allergenic plants pollen.

## 5. Conclusions

Our study demonstrates the urgent need for more coordinated efforts to evaluate biologic pollution in urban environments, to establish a national aerobiology network and to implement pollen monitoring in more centers from the country. It is important to develop further research projects and multidisciplinary collaboration with national and European institutions and specialists, in order to reduce the burden of pollen allergies and improve public healthcare.

## Figures and Tables

**Table 1:** Trees total monthly pollen grains/m^3^ air for five years (2014–2018).

Year	Pollen Species	March	April	May	June	July	Pollen Species	March	April	May	June	July
2014	Mulberry (*Moraceae*)		1247	51	5		Poplar (*Salicaceae*)	401		2		1
2015			456	18				198	49			
2016			41	1	12	21			19	28	1	
2017			466	40	3			34	148	2		
2018			135	39	2			5	79	5		
2014	Cypress (*Cupressaceae*)	115	7	2	2	1	Elm (*Ulmaceae*)	120	7			
2015		272	10	10		1		104	17			
2016		35	29	40	7	11			30	10		
2017		200	40	15	6	4		13	2			
2018			22	17	2	3			26			
2014	Alder (*Betulaceae*)	143	26				Birch (*Betulaceae*)	86	12	2		
2015		15	4			4		8	15	3		2
2016			31	1	3	2		47	14	4	3	3
2017		51	169					2	39	4		
2018			41						12			
2014	Linden (*Tilia*)		5	1	30	7	Platanus (*Platanaceae*)		28	2	1	
2015		1	2	7	22	5			29			
2016				6	22	17						
2017				5	24	3			85	4		
2018					14	6						
2014	Pinus (*Pinaceae*)			28	4	1	Quercus (*Fagaceae*)		100	4		1
2015			2	30	9	1		5	6	4	4	
2016		1	16	3	2	2		1	4			2
2017			12	41	8	2		35	50	12	2	3
2018			1	25	2	8			56	12		
2014	Fraxinus (*Oleaceae*)	70	30	10	9	1	Juglans (*Juglandaceae*)		15	16		
2015		80	52	44	2				6	4		
2016			17	9	16	7		1	5			
2017		30	55	23	4				20	2		
2018			54	20								

**Table 2:** Reported allergenic plants species in Bucharest (based on information from Professor Paulina Anastasiu, Botanical Garden, Bucharest).

Plant Family	Species	Spreading	Flowering Period
Grasses and Weeds	*Poaceae* (*Dactylis glomerata, Festuca rubra, Poa pratensis, Phleum pratense, Lolium perenae*)	Common	Spring–summer
	*Asteraceae* (*Ambrosia artemisiifolia* and *trifida*, *Artemisia vulgaris* and *absinthium*)	Very common	Summer–autumn
Trees	*Tiliaceae*	Very common	May–June
	*Juglandaceae*	Common	April–May
	*Fagaceae*	Common	April–May
	*Platanaceae*	Very common	April–May
	*Salicaceae*	Common	Early spring
	*Oleacea*	Common	April–May
	*Aceraceae*	Common	April–May
	*Pinaceae*	Common	Early spring
	*Ulmaceae*	Common	Early spring
